# Integration of Oral Health Into the Well-Child Visit at Federally Qualified Health Centers: Study of 6 Clinics, August 2014–March 2015

**DOI:** 10.5888/pcd13.160066

**Published:** 2016-04-21

**Authors:** Judith Bernstein, Christina Gebel, Clemencia Vargas, Paul Geltman, Ashley Walter, Raul I. Garcia, Norman Tinanoff

**Affiliations:** Author Affiliations: Christina Gebel, Paul Geltman, Ashley Walter, Raul Garcia, Boston University, Boston, Massachusetts; Clemencia Vargas, Norman Tinanoff, Boston University and University of Maryland School of Dentistry, Baltimore, Maryland.

## Abstract

**Introduction:**

Early childhood caries, the most common chronic childhood disease, affects primary dentition and can impair eating, sleeping, and school performance. The disease is most prevalent among vulnerable populations with limited access to pediatric dental services. These same children generally receive well-child care at federally qualified health centers. The objective of this study was to identify facilitators and barriers to the integration of oral health into pediatric primary care at health centers to improve problem recognition, delivery of preventive measures, and referral to a dentist.

**Methods:**

We collected and analyzed background data and data from structured observations and 39 interviews with administrators and staff at 6 clinics in 2 states, Maryland and Massachusetts.

**Results:**

Participants valued oral health across professional roles but cited limited time, lack of training and expertise, low caregiver literacy, and lack of shared medical and dental electronic records as barriers to cooperation. Facilitators included an upper-level administration with the vision to see the value of integration, designated team leaders, and champions. An administration’s vision, *not* structural determinants, patient characteristics, or geographic location, predicted the level of integration. Interviewees generated multilevel recommendations to promote delivery of oral health preventive measures and services during a well-child visit.

**Conclusion:**

Poor oral health contributes to health care disparities. Barriers to integrating dental care into pediatric medical practice at health centers must be overcome to improve oral health for children living in poverty, with a disability, at a rural address, or any combination of these. Implementation will require adapting delivery systems to support multidisciplinary collaboration. Strategies suggested here may point the way to enhancing children’s oral health.

**Medscape CME**

Medscape, LLC, is pleased to provide online continuing medical education (CME) for this journal article, allowing clinicians the opportunity to earn CME credit.

This activity has been planned and implemented in accordance with the Essential Areas and Policies of the Accreditation Council for Continuing Medical Education (ACCME) through the joint providership of Medscape, LLC, and *Preventing Chronic Disease*. Medscape, LLC, is accredited by the ACCME to provide continuing medical education for physicians.

Medscape, LLC, designates this journal-based CME activity for a maximum of 1 ***AMA PRA Category 1 Credit(s)™***. Physicians should claim only the credit commensurate with the extent of their participation in the activity.

All other clinicians completing this activity will be issued a certificate of participation. To participate in this journal CME activity (1) review the learning objectives and author disclosures; (2) study the educational content; (3) take the post-test with a 75% minimum passing score and complete the evaluation at http://www.medscape.org/journal/pcd; and (4) view/print certificate.

Release date: April 28, 2016. Expiration date: April 28, 2017.

**Learning Objectives**

Upon completion of this activity, participants will be able to:

1. Identify the most common chronic disease among children
2. Evaluate variables associated with higher rates of oral health integration in well-child care
3. Assess perceived barriers to oral health integration
4. Analyze recommendations to improve the integration of oral health in well-child care

**EDITOR**

Teresa Ramsey

Editor, *Preventing Chronic Disease*

Disclosure: Teresa Ramsey has disclosed no relevant financial relationships.

**CME AUTHOR**

Charles P. Vega, MD

Clinical Professor of Family Medicine, University of California, Irvine

Disclosure: Charles P. Vega, MD, has disclosed the following relevant financial relationships:

Served as an advisor or consultant for: Allergan, Inc.; McNeil Consumer Healthcare; Takeda Pharmaceuticals North America, Inc.

Served as a speaker or a member of a speakers bureau for: Shire Pharmaceuticals

**AUTHORS**

Judith Bernstein, MSN, PhD

Boston University Center for Research to Evaluate and Eliminate Dental Disparities at The Boston University Henry M. Goldman School of Dental Medicine; Boston University School of Public Health Department of Community Health Sciences, Boston, Massachusetts

Disclosure: Judith Bernstein, MSN, PhD, has disclosed no relevant financial relationships.

Christina Gebel, MPH

Boston University Center for Research to Evaluate and Eliminate Dental Disparities at The Boston University Henry M. Goldman School of Dental Medicine; Boston University School of Public Health Department of Community Health Sciences, Boston, Massachusetts

Disclosure: Christina Gebel, MPH, has disclosed no relevant financial relationships.

Clemencia Vargas, DDS, PhD

Boston University Center for Research to Evaluate and Eliminate Dental Disparities at The Boston University Henry M. Goldman School of Dental Medicine, Boston, Massachusetts; Department of Orthodontics and Pediatric Dentistry, University of Maryland School of Dentistry, Baltimore

Disclosure: Clemencia Vargas, DDS, PhD, has disclosed no relevant financial relationships.

Paul Geltman, MD, MPH

Boston University Center for Research to Evaluate and Eliminate Dental Disparities at The Boston University Henry M. Goldman School of Dental Medicine, Boston, Massachusetts

Disclosure: Paul Geltman, MD, MPH, has disclosed no relevant financial relationships.

Ashley Walter, MPH

Boston University Center for Research to Evaluate and Eliminate Dental Disparities at The Boston University Henry M. Goldman School of Dental Medicine; Boston University School of Public Health Department of Community Health Sciences, Boston, Massachusetts

Disclosure: Ashley Walter, MPH, has disclosed no relevant financial relationships.

Raul Garcia, DMD, MMSc

Boston University Center for Research to Evaluate and Eliminate Dental Disparities at The Boston University Henry M. Goldman School of Dental Medicine, Boston, Massachusetts

Disclosure: Raul Garcia, DMD, MMSc, has disclosed no relevant financial relationships.

Norman Tinanoff, DDS

Boston University Center for Research to Evaluate and Eliminate Dental Disparities at The Boston University Henry M. Goldman School of Dental Medicine, Boston, Massachusetts; Department of Orthodontics and Pediatric Dentistry, University of Maryland School of Dentistry, Baltimore

Disclosure: Norman Tinanoff, DDS, has disclosed no relevant financial relationships.

## Introduction

Early childhood caries (ECC), the most common chronic childhood disease (1), affects the primary dentition of children under age 6; it also affects their sleep, nutrition, and school performance (1). ECC is on the US health care agenda (2) because of its high prevalence (3) and associated health disparities (4,5), which are largely attributable to socioeconomic factors associated with race/ethnicity, poverty, or both (4,6). Approximately 77% of children on Medicaid have contacted a pediatric clinician within the past 6 months (7). Many of these children receive their care in federally qualified health care centers (FQHCs), which are already focused on prevention (8). The US Preventive Services Task Force recommends that all children receive fluoride varnish starting at the age of 6 months or first tooth eruption (9), but pediatric providers in FQHCs are often the only source of oral health education and preventive measures for vulnerable young people (10), according to multiple professional organizations (11–15). These organizations advocate an expanded role for pediatric primary care clinicians, including oral health counseling and referral by age 1 year to a dental home, defined by the American Academy of Preventive Dentistry as an ongoing relationship between a dentist and a patient (16). Having a dental home increases the likelihood of a subsequent dental visit (17).

A 2014 systematic review reported, however, that prevention of ECC has limited reach in community health settings (18), despite federal statutes requiring FQHCs to provide “primary health services” (Section 330), which includes “preventative dental services” in the definition of primary health services [42 U.S.C. §254(a)(1) and §254b (b)(1)(A)(i)(III)(hh)] (19). Recent studies confirm gaps in care and low screening and referral rates for children on Medicaid (20–22).

Unlike previous studies of challenges to oral health integration into pediatric well-child care (13), this study seeks specifically to understand gaps in oral health prevention services in FQHC pediatric care settings, with particular attention to barriers and facilitators, best practices, and recommendations elicited from staff.

## Methods

We selected clinics at 3 diverse FQHCs in Massachusetts and 3 in Maryland to cover a range of geographic locations (rural, small community, and urban), organizational structures, patient populations, workforce composition, and financial resources across a continuum of oral health integration into pediatric services. Variables predictive of the level of integration of dental and pediatric services were determined from the literature on oral health integration into pediatric primary care. Clinics were ranked by their number of integration variables: 4 or 5 advanced integration, 3 intermediate, and 0 to 2 minimal. Contextual, organizational and professional data were collected from August 2014 through March 2015 in 3 formats: background information, key informant interviews, and direct observation. This project was classified exempt by institutional review boards at Boston University, the University of Maryland, and the University of Baltimore, where interview data were collected.

The chief financial officers (CFOs) or their designees provided information on a formatted spreadsheet on aggregate patient demographics, geographic and catchment area information, number and types of clinic personnel, operating budget and sources of income, description of oral health activities (if any), use of electronic medical records (EMR), and level of oral health integration into the pediatric EMR.

The study protocol specified 42 participants, 7 from each FQHC, representing “decision makers, clinicians, and support personnel” (eg, FQHC clinic directors, medical directors, pediatric physicians, nurse practitioners, nurses, medical assistants, and dentists). Interviewers used a 45–60 minute semistructured interview via telephone to elicit relevance of oral health for general health, current practices, degree of integration and feasibility of oral health activities as part of pediatric primary care, recommendations for integration of oral health into pediatrics, perceived facilitators and barriers for integration, and potential strategies to remove barriers.

In both states, study investigators observed each site for 3–4-hours. We used a tested oral health direct observation checklist (13) that included items on physical plant, workflow and patient throughputs, clinic surroundings, patient registration procedures, medical records charting issues, referrals and tracking, oral health discussions during medical appointments, distribution and storage of oral health preventive products, and degree of coordination of medical and dental clinics (if colocated).

For background information, we compared the formatted spreadsheets using Microsoft Excel. For key informant interviews, we transcribed audio recordings. Two qualitative data analysts and a senior member of the research team at Boston University independently coded 3 key informant interviews and assigned initial codes by using inductive coding methods, and then met to discuss the proposed code list to resolve any differences in interpretation. A master code list was entered into NVivo 10 software (QSR International). Interrater reliability was assessed and re-coded independently until interrater reliability was satisfactory (κ coefficient greater than 0.70). The remaining interviews were coded, and new codes were discussed, defined, and added to the master list by the research team until the list reached saturation. Thematic analysis identified and enriched constructs specified in the literature and also allowed new ideas to emerge independently (23). For direct observations, these data were used to verify placement of the clinic on the continuum of integration and to verify clinical background.

## Results

Correspondence between the proportion of patients with Medicaid insurance, the amount of uncompensated care, and the existence of collaborations with schools of dentistry were particularly notable (Table 1). Similarities in patient demographics and clinic characteristics (Table 2) existed across all levels of integration in these 6 FQHCs. Differences in size of population served and in clinic operating budgets were apparent, but we found no correlation between the level of integration of oral care into pediatric well-child services (Table 1) and potential determinants such as clinic size (defined as number of patients served), length of time in operation, rate of staff turnover, patient characteristics, geographic location, operating budget, or colocation of dental and pediatric practices (Table 1). For example, the most under-sourced clinic was the most highly integrated (Clinic A); the clinic with the highest rate of private insurance (Clinic C) was least likely to provide pediatric patients with dental referrals; and a clinic with a colocated dental practice had little integration, despite being in the same building (Clinic Z).

**Table 1:** Levels of Integration of Oral Health Prevention and Services Into Pediatric Well-Child Care at 6 Federally Qualified Health Care Centers in Massachusetts or Maryland, 2015

Characteristic	Advanced Integration		Intermediate Integration		Minimal Integration	
	Clinic A	Clinic X	Clinic B	Clinic Y	Clinic C	Clinic Z
**Electronic medical record integration**						
Oral health template or flag in pediatric EMR	No	No	No	No	No	No
Access to pediatric and dental problem lists	Yes	No	No	No	No	No
Automated referral tracking	No	No	No	No	No	No
**Clinical practices**						
Formalized referrals for oral health (does not include self-referral)	Yes	Yes	Yes	No	No	Yes
Fluoride varnish application in pediatrics	No	Yes	No	Yes	No	No
Caries risk assessments (beyond visual inspection)	No	No	No	No	No	No
**Staff characteristics**						
Includes an oral health champion	Yes	Yes	Yes	Yes	Yes	No
Regular dental/pediatric training or meetings	No	Yes	No	No	No	No
**Clinic characteristics**						
Certified medical home	Yes	Yes	Yes	Yes	Yes	Yes
Colocation with dental clinic	Yes	Yes	Yes	No	No	Yes
**Partnerships**						
Networking with local dental practices if not colocated	NA	NA	NA	Yes	Yes	NA
Formal dental school partnerships	Yes	Yes	Yes	No	No	Yes
**Total number of integration variables**	4	5	3	3	2	2

Abbreviation: NA, not applicable.

**Table 2:** Patient Demographics and Structural Characteristics by Level of Integration of 6 Federally Qualified Health Care Centers in Massachusetts or Maryland, 2015

Characteristics	Advanced Integration		Intermediate Integration		Minimal Integration	
	Clinic A	Clinic X	Clinic B	Clinic Y	Clinic C	Clinic Z
**Pediatric clinic patients**						
**No. of patients**	7,462	6,515	10,480	9,824	534	11,460
**Age of patients**						
0–5 y	3,077	1,460	2,532	3,208	94	3,005
6–13 y	2,374	2,589	4,783	3,256	175	3,874
14–21 y	2,011	2,466	3,165	3,360	265	4,581
**Race/ethnicity, %**						
Non-Hispanic white	4.0	10.3	87.0	28.0	100	18.3
Non-Hispanic black	88.0	2.0	4.9	10.0	0	10.7
Hispanic	3.5	79.5	8.1	30.0	0	48.9
Asian	0.8	1.9	0	27.0	0	6.2
Other	3.7	6.3	0	5.0	0	15.9
**Limited English proficiency, %**	4.0	32.0	70.0	45.0	0	55.8
**Payer source, %**						
Private	9.0	3.1	6.0	16.0	30.0	11.0
Medicaid	87.0	96.1	75.0	82.0	50.0	85.0
CSHCN	0	0.6	9.0	0	0	0
Self-pay	4.0	0	10.0	2.0	20.0	4.0
**Clinics**						
Geographic location	Urban	Small City	Rural	Urban	Rural	Urban
Patient population size (catchment area), n	208,979	40,249	90,000	200,000	100,000	90,000
Years in operation, n	42	40	30	40	8	40
Operating budget, $, in millions	11.4	35.4	14.3	34.5	2.9	66.0
Uncompensated care, %	13.6	0	10–15	9	7	12.0
Years CMO in office	8	0.5	2	15	<1	4
Clinical staff turnover	Low	Low	High	High	High	Low

Abbreviations: CSHCN, children with special health care needs; CMO, chief medical officer.

We present relevant comments from 39 completed interviews by theme to describe barriers and facilitators to integration of oral health care into well-child visits. Each comment was identified by the key informant’s role and the level of integration in her or his work setting.

Perceptions of the importance of oral health were positive. Clinicians and administrators in all 6 clinics thought that oral health promotion for young children was crucial for a child to function normally and an important part of overall health care needs: “If you’re in pain . . . it’s hard to focus, and sometimes it’s hard to behave well. . . .[Y]ou can’t function the way you want to” [pediatrician, advanced integration].

Clinicians and administrators noted the consequences for children of limited access to restorative dental care: “A lot of the families . . . have a very simple screening at a school or [commercial dentistry] clinic. I think the parents . . . feel like they can check dental off the list . . .but the kids have not had . . . comprehensive dental care” [chief executive officer (CEO), advanced integration].

Pediatric clinicians described significant challenges for parents, such as competing demands on time and energy, difficulties obtaining dental care, and low oral health literacy. “I think it’s not important [to them] until it’s a problem, and then it’s very important” [pediatrician, advanced integration].

Clinicians described various barriers and gaps to including oral health in care delivery. In the clinical setting, time was a major challenge: “You’ve got a [patient] family. . . .They’re divorced, and the kids spend time in different places, and there’s smokers in the house, and they don’t eat right. They don’t have a flush toilet, and they’re playing hooky from school. After a while, how much can you fit into a 20-minute visit?” [chief medical officer (CMO), intermediate integration].

Administrators and clinicians spoke of concerns that oral health integration would overstep a pediatric clinician’s defined role and expertise: “Where we’re getting the challenge is not only the lack of [oral health] education . . . and sometimes the provider not being ‘comfortable,’ if you will, just because it’s not their area of expertise and they’re not trained a lot of times to look for certain things” [chief operating officer (COO), intermediate integration].

Both dental and pediatric clinicians felt going beyond a defined role could have potentially negative results for the patient by creating confusion or missing something: “Our exam is certainly not as thorough as the dental exam is going to be, and my biggest worry is the gum issues and the gum disease that we may not be picking up. . . .” [CMO, intermediate integration]. “They [pediatricians] could do a cursory look, but a cursory look does not always tell you what’s going on. So I always say, refer to the dental department” [dental director, advanced integration].

Many pediatric clinicians reflected on their lack of oral health training in pediatric residency programs or in the FQHC setting: “I don't think I’ve been to training at all. . . . I think in med school, we did some mild stuff, but no additional training in any technique” [physician, minimal integration].

Clinicians and administrators in more integrated clinics reported efforts at collaboration between dental and pediatric units, with regular staff in-services, presentations, or meetings, but multiple clinic priorities limited the amount of time available for cross-specialty communication and education. “We do have some staff meetings . . . like monthly. But, it seems like we’re busy trying to put out a fire as opposed to coming out with newer ideas for moving forward” [dentist, advanced integration].

Across all clinics, we found that none of the clinic administrators or pediatric clinicians were aware of federal regulations requiring integration of oral health into pediatric practice in FQHCs (19), and they were also not familiar with the *Bright Futures* oral health guidelines, issued by the American Academy of Pediatrics (AAP) in conjunction with the Maternal and Child Health Bureau (24), or the Protecting All Children’s Teeth (PACT) training program, which includes the AAP’s guidelines for oral health best practices (15).

When facilitators to integration of oral health into pediatric well child care were discussed, several interviewees approved of a collaborative approach to health care delivery, and recognized the impact of oral health on other medical conditions: “I mean, you can’t separate the mouth from the child and the dentist from the medical provider. It all needs to be integrated” [nurse practitioner, intermediate integration].

Staff in clinics with varying degrees of integration valued internal communication and working together on new or existing initiatives, and sharing resources to understand the context of a patient’s family life: “They may have a rapport with the family. They may know the family situation a lot better than me” [chief dental officer, intermediate integration].

Interviewees from some clinics also reported using outside resources, partnering with dental schools or outside private practices to increase access for their patients: “I’ve found that that is very helpful when the dental residents are here and they can be face-to-face with the patients. They come into all of our visits with us, and say, ‘If you don’t have a dentist, you can come see me upstairs’” [pediatrician, advanced integration].

Furthermore, there was general agreement that integration could happen only with leadership and vision at the top. Upper-level administrators’ involvement was seen as critical in setting the tone for clinic priorities and empowering clinical staff: “The administration has to buy in to the importance of pediatric oral health and has to make it a priority among all of the multiple priorities that we have” [senior VP of outreach, intermediate integration]

Several clinicians and administrators mentioned the importance of designating a team leader to promote oral health, and many also encouraged designating champions for oral health as team leaders: “Any transformation that happens within our practice, there is naturally one person that becomes the leader” [chief compliance officer, minimal integration].

In discussing system challenges, respondents noted numerous structural barriers to integrated health care delivery. EMR systems posed a significant barrier to integration in each of the clinics that were studied. At 4 of the 6 sites that were colocated, 2 different EMR programs were used for pediatrics and dental charting, with minimal communication between the 2 systems. Dental and medical providers reported tedious separate login procedures to gain access to patient records. Respondents, particularly at clinics with advanced integration, expressed a strong desire to have this cross-communication, because both systems report on a patient’s health. However, providers reported not knowing about the activities of their colleagues: “Well, I haven’t seen what happens on the dental side because they are not here at my site” [nurse practitioner, intermediate integration]. “If the dentist was concerned about nutritional status . . . we don’t often get that information. While we are all connected under the health center, we are sort of together but we are separate” [pediatric nurse, minimal integration].

Another major challenge noted was the lack of an oral health template, a referral system for dental services, and the capacity to track referral outcomes: “I hope they make the appointment, and then they go to the clinic and that’s where I lose it, unfortunately, in the loop” [nurse practitioner, intermediate integration]. “We’re trying to figure out a better way to get feedback from the local dentists as well . . . but we haven't really formalized the process for that” [pediatrician, intermediate integration].

There were also challenges related to state and federal reimbursement policy. Concerns were raised by clinic administrators that some states have bundled (one fee) billing for a well-child visit: “There is no additional revenue unless they go into the dental chair . . . . We get the same amount of money from Medicaid on every child visit” [president chief executive, intermediate integration].

Interviewees were asked to reflect on what changes would be needed to integrate oral health into routine pediatric care. Their recommendations are listed in the Box.

**Box. Recommendations of Key Informants From 6 Federally Qualified Health Care Centers in Massachusetts or Maryland, 2015**

Recommendation 1: Identify champions and foster leadership from the top of institutional hierarchies down to grass roots quality improvement committees.

Action Item 1.1: Widely disseminate evidence that supports inclusion of oral health promotion strategies in pediatric practice and relevant policy statements from professional organizations to federally qualified health center chief executive officers, chief medical officers, clinicians, and support staff.

Action Item 1.2: Review internal decision-making culture and structures to assess potential to be inclusive and conducive to change.

Action Item 1.3: Appoint 2 team leaders or oral health champions (1 from pediatrics and 1 from dentistry) who are in regular communication with upper-level management about progress and next steps for integration.

Action Item 1.4: Form an oral health committee, comprising many stakeholders involved in planning, implementation, and quality improvement.

Recommendation 2: Create an internal mechanism to reward health center staff champions, innovators, and oral health providers to develop and sustain integrated care within Centers; and establish external incentives for Centers that choose to implement integration of oral health into pediatrics within their health care systems.

Recommendation 3: Implement standardized, ongoing quality improvement measures.

Action Item 3.1: Evaluate documentation and ease of extraction of data about oral health procedures and prevention counseling, and remove barriers to data collection.

Action Item 3.2: Provide regular feedback to pediatric staff on oral health activities.

Recommendation 4: Provide greater opportunities for training pediatric staff in preventive oral health.

Action Item 4.1: Provide onsite workshops and links to useful oral health materials for all pediatric staff.

Recommendation 5: Increase funding for oral health in federally qualified health centers and increase awareness of funding opportunities.

Action Item 5.1: Increase funding opportunities to offer workshops on securing funding and using it to hire additional staff or expand dental-related activities, with a particular focus on connecting pediatric and dental efforts.

## Discussion

The climate for integration in FQHCs was mixed. The participants valued oral health as a part of a child’s overall health and well-being. They reported efforts toward dental and medical collaboration, as well as barriers that complicate or prevent these efforts. Interviewees nearly unanimously noted constrained time for well-child care, especially when caring for families with complex social needs. An FQHC could have colocation of dental and medical services, as did 4 of the 6 sites in this study, but results confirm other findings that colocation of services is only the initial step toward implementing interdisciplinary care (25). Interestingly, levels of oral health integration were not associated with the explanatory variables presented in Table 2.

This study demonstrated that efforts toward interdisciplinary team-based collaboration can be complicated by professional boundaries, sometimes referred to as “professional silos” (26), as well as challenges from professional values, beliefs, attitudes, customs, and behaviors (27). Respondents felt uncomfortable stepping out of their defined roles and scope of practice, as noted in a recent report by the DentaQuest Foundation (13). This lack of confidence could be closely related to a nearly unanimous lack of training in medical or dental school, continuing education, or professional development. Providers need further education in assessment to improve accuracy in visible examinations in well-child care, and in anticipatory guidance, as demonstrated by a lack of knowledge of oral health guidelines, such as recommendations on age of first dental visit (28,29). Interprofessional education improves core competencies (30) and could be a solution to overcoming this barrier.

As to systemic barriers, administrators and clinicians alike reported difficulty juggling multiple priorities and frustration with the lack of cross-communication in EMR systems. Finally, administrators and clinicians were unfamiliar with federal mandates and professional guidelines. Facilitators of integration were noted as well. Clinics with advanced integration, in particular, noted collaborative efforts between dental and pediatric staff. Clinic staff noted nearly unanimously that a committed vision from top administrators, designated team leaders, and oral health champions were necessary for meaningful change to occur. Key informants asked for ongoing quality improvement measures to highlight progress and identify gaps.

One surprising result from this study was that a clinic with low resources was among the most integrated in the study sample. The catchment area served by this clinic was recently named among the 3 poorest communities in its state, but the clinic’s lack of resources was balanced by a strong commitment to cross-disciplinary collaboration on the part of its CMO and leadership and supervision of integrative programs at each level of function, from central administration to program implementation to quality assurance. Similarly, integration did not necessarily follow colocation, as shown in Clinic Z, where a large, vibrant dental practice with strong administrative leadership interacted well with Family Practice clinicians, but not nearly as well with pediatricians. Through site observation, we noted that the signs of integration in family practice far exceeded the signs of integration in pediatrics.

Certain recommendations made by interviewees (Box) are conducive to immediate or short-term action, even for clinics with limited resources. Leadership vision statements, naming of champions, formation of an oral health committee, and naming of oral health topics for quality assurance can be taken without extra financial resources. When these actions demand allocated time, support from upper-level administration would help to ease the demand on staff schedules. Dissemination of best practices for oral health integration can be coupled with other clinic- or community-wide efforts for health promotion. Addition of an oral health template to the pediatric EMR can be approached by existing informational technology support staff. Systems changes, such as compatibility between pediatric and dental EMR systems, will be more difficult to overcome and may require dedicated financial resources for technical support.

This study has some limitations. First, although this study included a range of clinics, a larger sample would be necessary to ensure generalizability. Second, key informants were identified by clinic administrators, who may have chosen staff known to be knowledgeable about oral health or especially motivated.

FQHCs provide services for the children with the highest prevalence of early childhood caries, and thus have the greatest opportunity to deliver effective prevention measures. This study shows that pediatric administrators and clinicians in FQHCs collectively have the will but not yet the means to improve oral health and overall health for the children, families, and communities they serve. The strategies they suggested to remove barriers and challenges deserve further investigation and support.

## Post-Test Information

To obtain credit, you should first read the journal article. After reading the article, you should be able to answer the following, related, multiple-choice questions. To complete the questions (with a minimum 75% passing score) and earn continuing medical education (CME) credit, please go to http://www.medscape.org/journal/pcd. Credit cannot be obtained for tests completed on paper, although you may use the worksheet below to keep a record of your answers. You must be a registered user on Medscape.org. If you are not registered on Medscape.org, please click on the “Register” link on the right hand side of the website to register. Only one answer is correct for each question. Once you successfully answer all post-test questions you will be able to view and/or print your certificate. For questions regarding the content of this activity, contact the accredited provider, CME@medscape.net. For technical assistance, contact CME@webmd.net. American Medical Association’s Physician’s Recognition Award (AMA PRA) credits are accepted in the US as evidence of participation in CME activities. For further information on this award, please refer to http://www.ama-assn.org/ama/pub/about-ama/awards/ama-physicians-recognition-award.page. The AMA has determined that physicians not licensed in the US who participate in this CME activity are eligible for ***AMA PRA Category 1 Credits™***. Through agreements that the AMA has made with agencies in some countries, AMA PRA credit may be acceptable as evidence of participation in CME activities. If you are not licensed in the US, please complete the questions online, print the AMA PRA CME credit certificate and present it to your national medical association for review.

## Post-Test Questions

### Study Title: Integration of Oral Health Into the Well-Child Visit at Federally Qualified Health Centers: Study of 6 Clinics, August 2014–March 2015

#### CME Questions

1. You are seeing a 2-year-old girl for a well-child examination. Her parents report no health concerns, and you consider her risk for chronic illness. What is the ***most*** common chronic disease among children?
  - Obesity
  - Asthma
  - Anemia
  - Early childhood caries
2. Your examination of the patient's oral cavity demonstrates multiple caries and broken teeth. You ask the family about dental care, and they have not been to the dentist yet. Which of the following factors was most significant in the integration of oral health into the well-child pattern of federally qualified health centers in the current study?
  - Larger clinic size
  - Lower rate of staff turnover
  - Co-location of medical and dental services
  - None of the above
3. All of the following variables were considered barriers to integration of oral health into well-child care in the current study except:
  - General perception that oral health was a low priority
  - Challenges for parents in prioritizing oral health
  - Finding time during a clinic visit to address oral health
  - Poor training in oral health among medical providers
4. Which of the following statements regarding suggested means to improve the integration of oral health into well-child care in the current study is most accurate?
  - Strong involvement from staff was prioritized vs leadership from upper management
  - Champions of oral health were discouraged; oral health should be everyone's job
  - Stand-alone electronic medical records that separate dental and medical visits were preferred
  - External incentives for clinics that practice integration effectively should be established

**Table:** Evaluation

**1. The activity supported the learning objectives.**				
**Strongly Disagree**	** **	** **	** **	**Strongly Agree**
1	2	3	4	5
**2. The material was organized clearly for learning to occur.**				
**Strongly Disagree**	** **	** **	** **	**Strongly Agree**
1	2	3	4	5
**3. The content learned from this activity will impact my practice.**				
**Strongly Disagree**	** **	** **	** **	**Strongly Agree**
1	2	3	4	5
**4. The activity was presented objectively and free of commercial bias.**				
**Strongly Disagree**	** **	** **	** **	**Strongly Agree**
1	2	3	4	5
